# A Patient with Artifactually Low Serum High Density Lipoprotein Cholesterol Due to Waldenstrom Macroglobulinemia

**DOI:** 10.7759/cureus.2900

**Published:** 2018-06-30

**Authors:** Amir Shahbaz, Kashif Aziz, Muhammad Umair, Paria Zarghamravanbakhsh, Issac Sachmechi

**Affiliations:** 1 Internal Medicine, Icahn School of Medicine at Mount Sinai Queen Hospital Center, New York, USA; 2 Internal Medicine, Icahn School of Medicine at Mount Sinai Queen Hospital Center, West Hempstead, USA; 3 Endocrinology, Icahn School of Medicine at Mount Sinai Queen Hospital Center, New York, USA

**Keywords:** paraproteinemia, waldenstrom macroglobulinemia, hdl

## Abstract

When very low or undetectable high density lipoprotein (HDL)-cholesterol (HDL-C) is encountered in clinical practice, a paraproteinemia should be suspected in the absence of genetic or more obvious secondary causes. We reported a case of artifactually low HDL-C in a 68-year-old man with a past medical history of vitamin B12 deficiency. Lipid panel showed total cholesterol (TC) 144 mg/dl, triglycerides (TG) 79 mg/dl, HDL-C 5 mg/dl, and low density lipoprotein (LDL) 123 mg/dl. HDL-C, which was determined three years prior to this presentation was found normal. The patient was prescribed extended release nicotinic acid. Further workup performed showed the ratio of APO B/APO A1 0.36 and direct LDL 28 mg/dl. In the absence of genetic or more obvious secondary causes, we hypothesized that low HDL-C in this patient was due to paraprotein interference in vitro with the liquid homogenous HDL assay. Serum protein electrophoresis demonstrated normal IgG and IgA and an abnormally high IgM at 3510 mg/dl (57-266). A bone marrow biopsy revealed Waldenstrom macroglobulinemia. A diagnostic workup for an isolated low HDL-C unmasking the diagnosis of Waldenstrom macroglobulinemia has been rarely reported. Care must be taken when using the homogeneous method for direct measurement of HDL-C as artifactually undetectable HDL-C might result in the mismanagement of patients with paraproteinemia.

## Introduction

The inverse association between high density lipoprotein (HDL)-cholesterol (HDL-C) and risk of developing coronary artery disease is well known. Circulating monoclonal proteins may interfere with one or more laboratory tests performed using liquid-based automatized analyzers. Interference caused by paraprotein precipitation in a liquid homogenous HDL-cholesterol assay can lead to artifactually low HDL-C. Inaccurate measurement of HDL-C can lead to misclassification and unnecessary treatment. Here we presented a case of artifactually low measured HDL-C leading to a diagnosis of Waldenstrom macroglobulinemia.

## Case presentation

A 68-year-old man presented to our outpatient clinic for good health maintenance. He had a past medical history of vitamin B12 deficiency for which he was getting monthly parenteral therapy. A detailed history is taken and he reported that he had no fever, chills, diarrhea, cough, anorexia, weight loss, or rash. On physical examination, the heart rate was 85 beats/min and the blood pressure was 130/70 mmHg. The abdomen was soft, with normal bowel sounds and with no organomegaly. No peripheral edema was seen. No rash, petechiae, ecchymoses, oral lesions, or lymphadenopathy was found. The arms revealed no abnormalities and the legs were normal. Neurological examination was normal.

The levels of glucose, urea nitrogen, creatinine, calcium, phosphorus, and magnesium were normal; also the levels of total bilirubin, aminotransferase, and alkaline phosphatase were normal. Lipid panel showed total cholesterol (TC) 144 mg/dl, triglycerides (TG) 79 mg /dl, HDL-C 5 mg/dl, and low density lipoprotein (LDL) 123 mg/dl. Lipid panel done three years ago showed a HDL-C of 41 mg/dl. Further workup revealed serum apolipoprotein A1 (APO A1) 97 mg/dl (94-176), apolipoprotein B (APO B) 35 mg/dl (52-109), the ratio of APO B/ APO A 1 0.36 , lipoprotein A 19.0 nmol/l (<75), and direct LDL 28 mg/dl ( <130). Important laboratory investigations are given in Table 1.

**Table 1:** Laboratory results. *Abbreviations*: AST, aspartate amniotransferase; ALT, alanine aminotransferase; GGT, gamma glutamyl transferase; A PHOS, alkaline phosphatase; LDH, lactate dehydrogenase.

Tests	Results	Reference range
AST	20 U/l	2-40 U/l
ALT	18 U/l	2-50 U/l
GGT	15 U/l	0-40 U/l
A PHOS	109 U/l	30-115 U/l
LDH	129 U/l	90-225 U/l
Albumin	3.2 g/dl	3.5-5.0 g/dl
Total protein	8.7 g/dl	6.5-8.5 g/dl
Total cholesterol	144 mg/dl	150-230 mg/dl
Triglycerides	79 mg/dl	57-112 mg/dl
HDL	5 mg/dl	32-49 mg/dl
LDL	123 mg/dl	98-146 mg/dl
APO A1	97 mg/dl	94-176 mg/dl
APO B	35 mg/dl	52-109 mg/dl
APOB/APO A1	0.36	
Direct LDL	28 mg/dl	<130 mg/dl

 

The patient was treated with an incremental dose of extended-release nicotinic acid but his HDL remained low. The secondary causes of low HDL-C levels such as the use of androgens, progestins, cigarette smoking, obesity, low-fat diet, and drugs like beta-blockers were ruled out. This leads us to consider the less well-known but well-documented fact that monoclonal gammopathies have unusual specificity for apolipoprotein, and paraproteins may interfere with the measurement of HDL-C in some automated analyzers. To confirm our hypothesis we performed serum protein electrophoresis with immunofixation, which showed IgG 720 mg/dl (576-1782), IgA 116 mg/dl ( 59-484) and an abnormally high IgM at 3510 mg/dl (57-266). Bone marrow biopsy showed a paratrabecular and intertrabecular infiltrates of small noncleaved lymphocytes. Immunohistochemical studies showed B cell phenotype (CD 45, CD20 positive, and negative CD 3 and CD 10) consistent with Waldenstrom macroglobulinemia. He is being followed conservatively by hematology as has been stable thus far.

## Discussion

Extremely low serum HDL-C levels (<20 mg/dl) are associated with an increased risk of death, sepsis, and malignancy [1]. The primary causes of very low HDL-C levels are uncommon and they are the result of major genetic mutations of key steps in HDL metabolism. The secondary causes of severe HDL-C deficiency include hypertriglyceridemia, critical illness, androgenic anabolic steroids, and acquired lecithin cholesteryl acyltransferase deficiency and liver disease. Fairly rapid development of severe HDL-C deficiency in ambulatory subjects with previously normal HDL-C and triglyceride levels may occur with peroxisome proliferation-activated receptor agonist treatment or in patients with benign or malignant paraproteinemias [2]. Paraproteinemias are immunoproliferative disorders with lipoprotein abnormalities. We report a case of artifactually low HDL-C as measured with direct methods in a patient of monoclonal gammopathy. The observation of falsely low concentrations of HDL-C might result in the mismanagement of patients with paraproteinemia, as HDL-C concentrations are considered as a negative risk factor for cardiovascular diseases. Care must be taken when using the homogeneous method for direct measurement of LDL-cholesterol and HDL-C in patients with paraproteinemia [3]. Although it is difficult to predict which specimens cause interferences in automated analyzers [4], the problem can be solved by avoiding the presence of the proteins in the assay, performing the analysis using an alternative method or diluting out the interference [5]. Homogeneous assays show improved accuracy and precision in normal serum, and discrepant results exist in samples with atypical lipoprotein characteristics. Hypertriglyceridemia and monoclonal paraproteins are important interfering factors. A novel approach is the nuclear magnetic resonance spectroscopy that allows rapid and reliable analysis of lipoprotein subclasses, which may improve the identification of individuals at an increased coronary heart disease (CHD) risk. Apolipoprotein A-I, the major protein of HDL, has been proposed as an alternative cardioprotective marker avoiding the analytical limitations of HDL-C [6]. This patient had a normal HDL-C level three years ago, before the diagnostic work, ruling out genetic causes. None of the secondary causes like androgen, cigarette smoking, obesity low-fat diet, and drugs like probucol explain this low HDL-C. As the value of APO A1 and APO B were not co-relating with low HDL,suspicion of interference becomes obvious. The automated analyzer uses cholesterol esterase yielding hydrogen peroxide (H_2_O_2_) which is measured in peroxidized catalyzed reaction forming a dye measured photometrically. Because of interference of paraproteins, baseline observations are higher than normal; also the value calculated for HDL by subtracting initial from final observation is low [7]. This interference is prevented by removal of paraproteins prior to analysis of the sample or by dilution of the sample. The artifactually low HDL-C in this patient was caused by paraprotein interference in vitro with the liquid homogenous HDL assay but a diagnostic workup for an isolated low HDL-C unmasked the diagnosis of Waldenstrom macroglobulinemia.

## Conclusions

Both laboratorians and clinicians should be aware of the interferences in the clinical laboratory as the clinical consequences could be important. Techniques such as dilution, using solid-based assay or semi-quantitative electrophoresis if available should be employed to distinguish between pure in vitro artifact and real alteration. When a very low or undetectable HDL-C is encountered in clinical practice in the absence of genetic or more obvious secondary causes, a paraproteinemia should be suspected.
